# Central Committee's Bill

**Published:** 1919-07-12

**Authors:** 


### NURSE REGISTRATION.

### CENTRAL COMMITTEE'S BILL.

### Adjourned Debate in House of Commons.

### FRIDAY, JULY 4.

DEBATE resumed on amendment proposed on consideration, as amended (in the Standing Committee) (June 27):

CLAUSE 4.—(*Constitution and Appointment of Council.*)

(1) The Council shall consist of forty-two persons to be appointed or elected as follows:—

(*a*) Three persons to be appointed by the Privy Council;

(*b*) Four registered medical practitioners, two to be appointed by the Local Government Board for England, one for England, one for Wales, one by the Local Government Board for Scotland, and one by the Local Government Board for Ireland;

(*c*) Three registered medical practitioners to be appointed by the British Medical Association, one to be resident in England, one to be resident in Scotland, and one to be resident in Ireland.

Amendment proposed (June 27): To leave out the words:

(*a*) Three persons to be appointed by the Privy Council;

(*b*) Four registered medical practitioners, two to be appointed by the Local Government Board for England, one for England, one for Wales, one by the Local Government Board for Scotland, and one by the Local Government Board for Ireland;

(*c*) Three registered medical practitioners to be appointed by the British Medical Association, one to be resident in England, one to be resident in Scotland, and one to be resident in Ireland; and to insert instead thereof the words,

" namely, one person to be appointed by the Privy Council, one person by the British Medical Association, two persons by the College of Nursing, Limited, one person by the Royal British Nurses' Association, and thirty-seven nurses by election on the part of all nurses duly registered, the first election to take place on the expiration of two years from the passing hereof."—[*Lieut.-Commander Astbury.*]

Question again proposed,

"That the word 'Three' stand part of the Bill."

**Major Nall:** I beg to move, "That the debate be now adjourned." I think a good many hon. Members will feel that with only an hour to spare we cannot adequately consider the many amendments on the Paper dealing with this important question. I am prompted to take this course by the statement which was made by the Minister of Health last Friday, when he very clearly explained the position.

**Major Barnett:** The hon. and gallant Member spoke last week on the amendment now before the House. Is he in order in moving the adjournment?

**Mr. Speaker:** If the hon. Member spoke on the amendment on the last occasion he would not be entitled to speak again on the amendment now.

**Major Nall:** I am moving "That the debate be now adjourned."

**The Speaker:** That is speaking to this amendment, and the hon. member is disqualified from doing so.

**Major Nall:** May I move "That further consideration be now adjourned?"

**Mr. Speaker:** No. What is now before the House is the amendment, and on that the hon. member has already spoken.

Amendment negatived.

**Major Barnett:** I beg to move in paragraph (*b*), after the word "and" to insert the words "one by the."

**Major Nall:** May I now move the adjournment of the further consideration of the Bill?

**Mr. Speaker:** Yes.

**Major Nall:** I beg to move "That the debate be now adjourned."

**The Minister of Health,** speaking last week, said that there had been conferences "with the view of discovering whether a sufficient common measure of agreement could be reached by which we could obtain a Bill which would give effect to the registration of nurses which I think by common consent is regarded as most necessary and desirable. I am sorry to say that the results of these conferences have convinced me against my will that such agreement is not obtainable. I think it arises from the fact that while everybody agrees that the registration of nurses is desirable and necessary, it was quite clear in the conferences that those who are interested in the two Bills were not by any means agreed, nor were they likely to agree as to what was implied by registration."

"the controversy appears to have been unfortunately mixed up with personal and sectional issues which cannot be reconciled."—[OFFICIAL REPORT, Friday, June 27, 1919, cols. 397 and 398, Vol. 117.]

He explained that if the promoters of this Bill would allow it to drop and the promoters of the opposing Bill in another place would withdraw that Bill, the Government were prepared to bring in what they considered was a fair Bill, and to deal with this great question unbiased by any of the sectional interests which have unfortunately divided the nursing profession. I am authorised to say by the promoters of the Bill in another place—although when I originally spoke last week I explained that I was not pressing an amendment on behalf of the College of Nursing —that I have received a letter from the Chairman of the College of Nursing in which he says:

"I should be glad if you would take an opportunity of stating on behalf of the College that on the Government undertaking to introduce a Registration Bill we will at once withdraw the College Bill."

That is a definite undertaking by the promoters of the Bill in another place, and I suggest that in view of the Government promise it will be much fairer to the nursing profession to accept the right hon. gentleman's offer and to have a proper Government Bill dealing with the question on national lines. As the promoters of the Bill now under consideration refused last week to accept the right hon. gentleman's offer, and there is very little time left this afternoon to consider the amendments, I beg to move "That further consideration of this Bill be now adjourned."

**Mr. Hailwood:** I beg to second the motion. As long as there are differences of opinion so strongly held, and the whole House wishes to do the best it can in the interests of the nurses, I think the best thing we can do is to adjourn the debate until the two sections of nurses are in agreement as to the right course to pursue.

**Major Barnett:** The mover of the adjournment has said that his object is that the Bill should be "allowed to lapse." The hon. and gallant member who moved the amendment was careful to say at the beginning of his speech that he was not out to wreck the Bill, but it was an amendment which went to the whole root of the Bill, which took away a very large and vital part of the measure, which affected the constitution of the General Nursing Council and substituted a perfectly new Council which may have been better or worse.

**Major Nall:** If there was any chance of considering the amendments adequately I should be quite prepared to join my hon. and gallant friend in doing so. I made the remark "allowed to lapse" because I understood that that was the only thing that will happen.

**Major Barnett:** The right way is not to move wrecking amendments. There was every opportunity last week of

**Nurse Registration**—(*continued*).

obtaining the Report stage of this Bill. The amendment by the hon. and gallant member for West Salford, supported by my right hon. and gallant friend, was backed by arguments of the most astounding character. One statement was that 14,000 nurses constituted a third of all the nurses of the country. If he will look at the census he will find there are 80,000 nurses in the United Kingdom, and yet he said 14,000 represented two-thirds of the nurses in the kingdom. It was by arguments of that kind that the hon. and gallant gentleman opposed. He spoke, too, of democracy, and said this was not a democratic Bill, and yet he advocated a Nursing Council, that might not have necessarily a single nurse upon it, and still he prates about democracy. The Bill, which was read a second time in another place, was on the basis that it could be compared by the other House with this Bill, and now the friends of the Nursing College come down here with an organised attempt and probably a successful attempt to prevent this Bill getting to the House of Lords at all. If they have their way I wish it to be quite clear to the country who has wrecked this Bill, which embodies a principle so ardently desired by the nursing profession, and a principle which has been adopted twice in this House, and which had a good chance of getting on to the Statute Book in this Bill, but which has been prevented by a number of members who pay lip-service to the principle of registration and then strain every nerve to prevent it being adopted by the House. When hon. members put down five or six pages of amendments to a Bill at this stage they have no desire to see the Bill go through. As to the pledge given, I have no doubt the Government in its own good time will introduce a measure, but if it had not been for the time taken up by wrecking amendments and motions for adjournment, it would have been possible to have dealt with all the real amendments promised in the Committee stage, and passed the Bill here with a good chance in another place of some agreement being arrived at. Whether the Government, in face of the spirit shown by some hon. members here, is going to be brave enough to bring forward a measure for the registration of nurses remains to be seen. I hope the Minister of Health will have the courage of his convictions, and will bring forward such a measure.

**Major Astor:** Oh, yes.

**Major Barnett:** We have now a renewed assurance that the measure will be forthcoming. I quite agree with the hon. member who moved the amendment that it is impossible, even with every good will, to deal with all the amendments now. Under those circumstances, having made my protest, and made it quite clear that the failure of this Bill is none of my doing, but the result of organised and concerted opposition, I can sit down and look forward to the near day when the ardent desire of the nurses for State Registration and for the placing of their profession upon a sound, self-respecting basis, will be carried into effect.

**Mr. Pemberton Billing:** I congratulate the hon. member who has just sat down on completely clearing the character of the nurses of the country, but it is of more importance that we should leave this question with a perfectly clear mind. My experience goes to show that a Bill which has not the official support of the Government has very little chance of getting on the Statute Book. The exception which we have seen just now must be taken as an exception, and I think it was rather unfortunate that the last speaker should assume that because anyone dare to criticise or offer amendments they were necessarily obstructionists. The Minister of Health has given us an undertaking that this matter shall form the subject of a Bill to be introduced by the Government, and I think it would be far better for hon. members to await such a Bill, and when it comes before the House to see that the interests of the nurses are protected. There is no doubt about it that so keen is the feeling on this subject for the Government to take this matter up that they will do it. Surely there is nothing more required, and I trust that there will be no more discussion now, but that we shall be able to get down at the earliest possible moment to the proposals of the Government, criticise them, it may be, and introduce such amendments to safeguard the nursing industry throughout the country as may be found necessary.

**Sir Courtenay Warner:** There has been a great deal of controversy as to which nurses should vote, but there is one thing I hope will be put into the Government Bill when it comes, and that is that the vote of the nurses shall not depend on registration entirely, that women who have gone in and done a year's probation work in a hospital shall have a vote equally with those who have been registered. At the present moment, what are called probationers in hospitals are practically in the position of the slaves of the nurses, and they are the class that want more protection than anyone else in the nursing world. Some hon. members will know, having had daughters nursing in hospitals during the war, the sort of thing that goes on in hospitals, and that the voice of the untrained nurse or the nurse who has not been completely trained is absolutely unheard and absolutely unrepresented in either of these Bills, and I hope something will be done to give them representation in some way. I think it is a pity that this discussion should not go on, because the further we get with it the more likely is the Bill to get through when the Government brings it in.

Question put, and agreed to.

Debate to be resumed upon Friday, July 25.

